# Gender-Inclusive Language in Public-Facing Labor and Delivery Web Pages in the New York Tristate Area: Cross-Sectional Study

**DOI:** 10.2196/53057

**Published:** 2025-01-06

**Authors:** Sarah Mohsen Isaac, Mark Dawes, Emily Ruth Howell, Antonia Francis Oladipo

**Affiliations:** 1 Hackensack Meridian School of Medicine, 123 Metro Blvd, Nutley, NJ, 07110, United States, 1 7248419463; 2 Cornell University, Ithaca, NY, United States; 3 Department of Obstetrics and Gynecology, Hackensack University Medical Center, Hackensack, NJ, United States; 4 Division on Maternal-Fetal Medicine, Department of Obstetrics and Gynecology, Hackensack University Medical Center, Hackensack, NJ, United States

**Keywords:** OBGYN, transgender, nonbinary, pregnancy, maternity, transmasculine, observational study, gestational, perinatal care, communication, labor, USA, United States, New York City, sexual orientation, inclusion, parents, obstetrician gynecologist, delivery

## Abstract

**Background:**

Transgender and nonbinary (TGNB) individuals are increasingly intentionally becoming pregnant to raise children, and hospital websites should reflect these trends. For prospective TGNB parents, a hospital website is the only way they can assess their safety from discrimination while receiving perinatal care. Cisnormativity enforced by communication gaps between medical institutions and TGNB patients can and has caused delays in receiving urgent care during their pregnancy.

**Objective:**

The aim of this study was to evaluate the current prevalence of gender-inclusive terminology among labor and delivery services in the New York tristate area.

**Methods:**

The labor and delivery web pages of 189 hospitals from New York, New Jersey, and Connecticut were examined for gender-inclusive language. “Fully inclusive” websites explicitly acknowledged lesbian, gay, bisexual, transgender, queer, intersex, and asexual plus other gender- and sexual-oriented (LGBTQIA+) parents, “inclusive” websites did not use gendered terminology for parents, and “noninclusive” websites used gendered terms at least once in the text reviewed. The hospitals’ web pages were further stratified by Healthcare Equality Index scores and population classifications defined by the 2013 National Center for Health Statistics Urban-Rural classification given to the county that each hospital was located in.

**Results:**

Of the 300 hospital websites reviewed, only 189 websites met the criteria for inclusion. Overall, only 6.3% (n=12) of labor and delivery web pages were “inclusive” or “fully inclusive.” No geographic areas (*P*=.61) or Healthcare Equality Index scores (*P*=.81) were associated with inclusive or fully inclusive language.

**Conclusions:**

Hospitals need to use inclusive language to help TGNB people identify hospitals where their existence and needs are acknowledged and thus feel more comfortable in their transition to parenthood.

## Introduction

Within the last 2 decades, as transgender and nonbinary (TGNB) people have gained greater legal and social recognition, those in the TGNB community who want to become pregnant have become increasingly more common, as have the number of families with same-sex, transgender, or nonbinary parents [1]. Despite this trend, the field of medicine has mostly maintained the heteronormative model of a “mother” and “father” as opposed to a more fluid, freeform reality that accompanies the rise of lesbian, gay, bisexual, transgender, queer, intersex, and asexual plus other gender- and sexual-oriented (LGBTQIA+) couples who raise children.

As pregnant TGNB people attend numerous prenatal visits, they make repeated contact with heterosexist health care systems without the ability to hide their transgender status. Pharr [2] explains that heterosexism is not an active form of discrimination but rather “a belief that the world is and must be heterosexual.” According to the heterosexist worldview, every couple contains—or should contain—only 2 gender-conforming people of the opposite sex [3].

Heterosexism works with homophobia to make health care inaccessible for LGBTQIA+ populations. Current literature has found that these parents and couples are often invalidated and marginalized throughout the health care process through obstacles like registration forms, comments from ancillary staff, and physicians who are unprepared to deal with LGBTQIA+ couples [4-6]. These experiences can take a deep mental and emotional toll—doubly so during the sensitive transition to parenthood [7].

Beyond these mental health impacts, the invisibility of TGNB parents can negatively affect pregnancy outcomes. LGBTQIA+ patients were less likely to trust providers and divulge important medical information when they received heteronormative medical treatment [8,9]. Patients may also experience delays receiving urgent pregnancy care due to the systematic heterosexism built into the health care system.

For example, Parker et al [6] discuss the experiences of a transmasculine patient who was delayed in seeing the doctor because the receptionist argued that he was not the intended person. These patterns are not limited to individual heterosexist health care workers but also integrated into hospital software. Berger et al [10] describe another scenario where a transmasculine patient’s care was delayed, this time because the hospital’s electronic medical record required that he reregister as female to document the pregnancy, regardless of his actual gender identity. A delay in care to circumvent cisnormative systems can be dangerous for all pregnant TGNB people. Ultimately, discrimination has often necessitated that prospective TGNB parents discern a “safe” hospital before seeking care.

Historically, LGBTQIA+ people have relied on word of mouth from their personal social circles to find safer health care [11]. However, younger LGBTQIA+ people, especially ones without LGBTQIA+ support networks, also rely on the internet to search for health information and providers that are inclusive of LGBTQIA+ people [12,13]. This vetting of hospitals, along with the increase of patient choice and consumerism for perinatal care in general, has prompted hospitals to advertise their unique benefits, such as low cesarean rates, “baby-friendly” designations, and private rooms [14-19]. Hospitals have tailored their advertisements for other demographics around them, but there is a dearth of literature showing how hospitals advertise their services for LGBTQIA+ populations, who rely on publicly available information to find inclusive care and preserve their health and safety [20].

Purdie-Vaughns et al [21] point to purposeful word choice as one safety cue that, when recognized, signals protection from identity-based discrimination. Hospitals might therefore attract pregnant LGBTQIA+ parents by crafting more inclusive obstetrical web pages. These pages could signal inclusivity by explicitly referencing LGBTQIA+ care or by avoiding gender-exclusive language like “mother and baby,” “mom,” or presumptive she/her pronouns for parents. Through the words chosen on these public-facing web pages, hospitals thus enable parents to choose to give birth in places where their existence is actively supported during the physically dangerous and psychologically difficult transition to parenthood.

The states surrounding New York City—New York, New Jersey, and Connecticut—boast an exceptionally high population density of LGBTQIA+ individuals, who make up between 3% to 5% of the total adult population [22]. This geographical region is viewed as more inclusive towards LGBTQIA+ people than average, so hospitals may have more incentive to provide inclusive care [23,24]. This study aims to evaluate the current prevalence of LGBTQIA+ inclusion and gender-inclusive terminology among labor and delivery (L&D) service web pages in the New York tristate area.

## Methods

### Study Design

The targeted words used to assess gender-inclusiveness for this study were largely adapted from the Jennings et al [25] study of gender-inclusive language on National Health Service websites.

The official public-facing obstetric web pages of nonfederal, short-term, acute-care hospitals from Connecticut, New Jersey, and New York were analyzed (n=300). Hospitals without L&D services or web content describing these services were excluded (n=189). Websites were reviewed from late November 2022 to January 2023.

Hospitals were categorized by state, 2013 National Center for Health Statistics Rural-Urban classification, and Healthcare Equality Index (HEI) score. The National Center for Health Statistics Rural-Urban classification is a tool used to identify urban and rural areas of the United States. It was used to analyze any association between urbanization and hospital-based inclusiveness of LGBTQIA+ people. The HEI score is the national LGBTQIA+ benchmarking criterion that assesses health care facilities’ policies and practices regarding equity and inclusion of LGBTQIA+ patients, visitors, and employees. It was also used to identify if there was any association between a hospital’s publicly perceived LGBTQIA+ inclusivity and word choice on the web pages.

For each hospital, at least 1 web page was examined alongside up to 2 additional pages as supplementation for language analysis. The gendered language used was recorded and analyzed by a single reviewer. The complete L&D-related text was analyzed and the types of gendered language used were recorded. (Explicit discussion of related services, such as chestfeeding, within the same site was excluded.) Any non–gender-inclusive descriptors for the name of the building or third-party services were also excluded from analysis, as these are often not controlled by hospital administration.

### Language Analysis

Each web page was reviewed independently by the chief reviewer to minimize any discrepancies. Each hospital’s L&D web page was rated as “fully inclusive,” “inclusive,” or “noninclusive.” “Fully inclusive” websites explicitly acknowledged LGBTQIA+ or TGNB parents. “Inclusive” websites did not use gendered terminology or pronouns for prospective parents. “Noninclusive” websites used the terms “woman” or “women”; “mom” or “mother”; other terms for women; “father” or “dad”; or she/her pronouns at least once in the text reviewed.

### Statistical Analysis

Categories were analyzed using *χ*^*2*^ tests presented as frequencies with percentages. *P* values <.05 were considered statistically significant, and all tests were 2-sided.

### Ethical Considerations

Ethics and insitutional review board approval were not required since the study did not include human or animal subjects and all data were collected from publicly available websites.

## Results

Of the 300 hospital websites reviewed, 111 hospital websites did not have a L&D web page or did not have content describing their L&D services (Multimedia Appendix 1). Of the remaining 189 websites analyzed, 12 (6.3%) of them used fully inclusive or inclusive language (Table 1). Only 1 hospital (0.5%) was considered fully inclusive because it acknowledged “same-sex” couples in its L&D content. The most common noninclusive terms used were “mom” or “mother” (n=166, 87.8%) and “woman” or “women” (n=94, 49.7%). No geographic areas (*P*=.61) or HEI scores (*P*=.81) were associated with inclusive or fully inclusive language (Tables2^4^).

**Table 1.** Labor and delivery web pages that used each type of language (N=189).

	Fully inclusive, n (%)	Inclusive, n (%)	Noninclusive, n (%)	Total, n (%)
Websites	1 (0.5)	11 (5.8)	177 (93.7)	189 (100)
Language used				
woman or women	1 (1.1)	0 (0)	93 (49.2)	94 (49.7)
mother or mom	0 (0)	0 (0)	166 (87.8)	166 (87.8)
she/her (parent)	0 (0)	0 (0)	42 (22.2)	42 (22.2)
she/her (staff)	0 (0)	0 (0)	9 (4.8)	9 (4.8)
synonyms for women (ladies, etc)	0 (0)	0 (0)	1 (0.5)	1 (0.5)
father or dad	0 (0)	0 (0)	33 (17.5)	33 (17.5)

**Table 2.** Summary of HEI^a^ scores and gender-inclusive language used on L&D^b^ web pages .

	Fully inclusive (n=1), n (%)	Inclusive (n=11), n (%)	Noninclusive (n=177), n (%)	Total (N=189), n (%)
HEI score=100%	0 (0)	5 (45.5)	60 (33.9)	65 (34.8)
HEI score <100%	0 (0)	1 (9.1)	25 (14.1)	26 (13.9)
HEI score not applicable	1 (100)	5 (45.5)	92 (52)	98 (52.4)

a HEI: Healthcare Equality Index.

b L&D: labor and delivery.

**Table 3.** Summary of gender-inclusive language used on labor and delivery web pages and population data.

	Fully inclusive (n=1), n (%)	Inclusive (n=11), n (%)	Noninclusive (n=177), n (%)	Total (N=189), n (%)
Large central metro	0 (0)	6 (54.5)	53 (30.6)	59 (31.2)
Large fringe metro	0 (0)	3 (27.3)	65 (37.6)	68 (36)
Medium metro	1 (100)	1 (9.1)	31 (17.9)	33 (17.5)
Small metro	0 (0)	0 (0)	7 (4)	7 (3.7)
Micropolitan	0 (0)	1 (9.1)	17 (9.8)	18 (9.5)
Noncore	0 (0)	0 (0)	4 (3.9)	4 (2.1)

**Table 4.** Examples of suggested gender-inclusive language [19,25].

Non–gender-inclusive language	Gender-inclusive language
“Mothers”	“Birthing parents” OR “women and birthing parents”
“Pregnant woman”	“Pregnant patient” OR “pregnant person”
“Mother and baby unit”	“Maternity unit” OR “birthing unit”

## Discussion

### Principal Findings

These results demonstrate that there is a large barrier for TGNB parents to search for and identify potentially inclusive pregnancy care. Out of the 12 inclusive and fully inclusive L&D web pages, 92% were inclusive not because they included gender-additive language or LGBTQIA+ topics but rather because they omitted the pregnant person’s gender altogether by addressing the reader in the second person. The websites may have been inclusive not by intention but by coincidence. In stark contrast, there are multiple private reproductive endocrinology and infertility clinics that specifically target LGBTQIA+ couples using specific gender-inclusive language on their websites [26]. TGNB parents who are accustomed to a purposefully inclusive experience during their fertility journey and early pregnancy may be caught off guard by the sudden invisibility of their identities as they progress further through their pregnancy.

Interestingly, none of the hospitals that are acknowledged for their excellence in LGBTQIA+ care in other specialties discussed serving prospective TGNB parents for L&D care on their websites. This likely reflects a wider societal trend of “repronormativity,” by which society at large does not recognize reproductive sex between TGNB parents as possible or legitimate [27].

The accessibility of websites and web-based platforms is important for TGNB people to find services and connect to similar parents. Our above findings suggest less than 10% of hospitals use gender-inclusive language when representing their services. Thus, TGNB parents who are not connected to a wider LGBTQIA+ community may struggle to find inclusive prenatal care and delivery services due to the lack of representation.

### Limitations

The use of multiple surrogate end points may limit this study. The analyzed web pages, while used as a proxy for the culture in L&D departments, may not fully represent institutional attitudes and practices once parents start using their providers. This is exacerbated by delays between institutional attitude changes and hospital website updates. HEI scores were ineffective in predicting the LGBTQIA+ inclusivity of hospitals’ web pages because HEI scores are determined purely through institutional measures like nondiscrimination policies; they do not directly address subtler, underlying heterosexism that hopeful TGNB parents try to avoid in their health care. Finally, the methodology used in this study makes it impossible to establish a causal link between gendered terminology and the quality of LGBTQIA+ inclusive care.

Additionally, it is important to note that individual TGNB people may feel varying levels of dysphoria around maternal terms; some TGNB parents may not consider the words “mom” or “mother” to be exclusively for women. However, using gender-inclusive language and terminology is an important step towards providing a more welcoming and inclusive environment for TGNB parents, regardless of those individuals’ personal dysphoria triggers.

### Conclusions

Using gender-inclusive language and terminology is the first step towards providing a more welcoming and inclusive environment for pregnant TGNB parents. Hospitals that want to be recognized as more inclusive towards LGBTQIA+ people can integrate gender-additive language into their L&D web pages (eg, “mothers and birthing parents”) rather than omit mentions of gender entirely [28]. US hospitals should consider expanding this language to meet the needs of a growing group of people who are having children. Future research should be done including LGBTQIA+ patient advocate groups on the use of inclusive language within health care providers’ obstetrical and gynecology departments, specifically in L&D units and on how this language impacts TGNB parents’ health outcomes and rapport with physicians.

## Supplementary material

10.2196/53057 Multimedia Appendix 1 List of hospitals reviewed for analysis, along with a list of hospitals where labor and delivery pages were missing for various reasons.

## Abbreviations

HEI: Healthcare Equality Index
L&D: labor and delivery
LGBTQIA+: lesbian, gay, bisexual, transgender, queer, intersex, and asexual plus other gender- and sexual-oriented identities
TGNB: transgender and nonbinary
